# ‘Facts that are declared proven’: sexual violence, forensic medicine, and the courtroom in early Francoist Spain.

**DOI:** 10.1080/09612025.2023.2197791

**Published:** 2023-04-16

**Authors:** Stephanie Wright

**Affiliations:** Department of History, Lancaster University, Lancaster, UK

**Keywords:** Francoism, Spain, forensic medicine, rape, courts

## Abstract

This article examines the Spanish court system as a site for the secondary victimisation or ‘second rape’ of sexual assault victims under the right-wing, Catholic dictatorship of Francisco Franco in Spain. Medical evidence enjoyed a high level of prestige as a modern and ‘objective’ arbiter of truth in Francoist Spain, precisely because of widespread recognition of the legal system’s corruptible nature. As such, contemporary court records reveal how victims in fact sometimes sought out medical examinations, even before reporting sexual crimes to law enforcement. However, the discretional nature of the Francoist legal system, heavily reliant on character references, allowed investigating judges to exploit the ambiguities of medical evidence to fit their vision of who constituted the legitimate ‘victims’ and ‘perpetrators’ of sexual violence. Medical forensic evidence therefore served an important purpose in Francoist rape trials; this was not the pursuit of justice or reparations for victims, but rather to reinforce conservative, patriarchal societal structures while providing a veneer of legitimacy to an otherwise distrusted legal system.

On the 18 July 1936, a group of military rebels launched a coup against the democratically elected Second Spanish Republic. The civil war which subsequently engulfed the country would lead to the imposition of the Catholic, military dictatorship of Francisco Franco, a regime which would rule over Spain until the 1970s. The extent to which the advent of Francoism reconfigured Spanish society has been the subject of countless studies in recent decades. In particular, historians of gender have pointed to the ways in which the regime redrew the boundaries of acceptable female morality, which shaped—or rather restricted—the public and private lives of Spanish women.^1^ Most recently, one of the fields within the historiography on modern Spain which has garnered increasing attention is the perpetration of sexual violence during the Civil War and the post-war period, and whether or not we can consider rape to have constituted a ‘weapon of war’ in the Spanish case.^2^ This article builds on these recent innovations, adding an extra dimension to such discussions by considering the experiences of victims beyond the initial attack itself. More specifically this research seeks to understand how the broader socio-political and legal context of Francoism shaped court cases linked to sexual crimes during the war and post-war period.

The question of evidence has historically posed challenges for women seeking justice for sexual crimes. In the present day, many victim-survivors who do report acts of sexual harm find that they undergo a secondary process of victimisation or ‘second rape’, in which victim-sceptic social, legal and medical attitudes compound feelings of shame, stigma, and trauma.^3^ Prior to the advent of forensic medicine in the nineteenth century, prosecutions for rape largely depended on the presence of eye-witnesses as well as the respective reputations of the victim and perpetrator.^4^ Over the course of the twentieth century, forensic doctors came to play an increasingly important role within the prosecution of sexual crimes, and medical forensic testimony came to be regarded as the ‘gold standard’ of evidence in rape trials, given the belief that it could bring scientific impartiality to proceedings.^5^ As such, during the modern period the body—usually that of the victim—came to be regarded as a living crime scene, often subjected to invasive medical forensic examination.

Spain was not marginalised from these developments, and forensic medicine began to coalesce as a discipline in the country from the nineteenth century.^6^ Meanwhile, recent interdisciplinary scholarship has thrown serious doubts on the utility of medical forensic evidence in the policing of sexual crimes, which is often misunderstood in the courtroom, can serve to reinforce rape myths, and ultimately offers little succour to the prosecution’s case.^7^ This article will contribute to this scholarship by examining the impact of Spain’s authoritarian political system on the forensic examination of rape victims, exploring the function of forensic medicine within Spain’s turbulent political context. In Spain, recourse to supposedly ‘modern’ medical knowledge ran parallel to a discretional legal tradition heavily reliant on character references, which were easily exploited within the dictatorial context. As such, investigating judges were able to mobilise witness testimony to exploit the ambiguities of medical evidence in order to reinforce their vision of who constituted the legitimate ‘victims’ and ‘perpetrators’ of sexual violence. This article will argue that medical forensic evidence therefore served an important purpose in Francoist rape trials; this was not the pursuit of justice or reparations for victims, but rather to reinforce conservative, patriarchal societal structures while providing a veneer of procedural objectivity to an otherwise distrusted legal system.

This argument will be developed through an assessment of academic forensic understandings of ‘virginity’ in 1930s-40s Spain, followed by an analysis of forensic ‘overreach’ in the courtroom, and a discussion of the manipulation of medical evidence by Spanish judges and court officials. While this article draws partially on civilian rape cases, particular attention is given to sexual crimes tried by the military courts. Under the dictatorship, the military court system—which generally addressed crimes committed by active and reservist armed forces personnel—came to have an increasing influence over the policing of sexual crimes.^8^ This was due both to the general expansion of the military courts’ jurisdiction with the advent of Francoism, as well as the large numbers of men who remained attached to the armed forces during and in the years following the Civil War.^9^ At the same time, as others have pointed out, the very experience of military mobilisation—particularly the misogynistic homosocial culture which characterised the armed forces—contributed to increased incidences of rape, thereby rendering it more likely that sexual violence would come to the attention of the military courts.^10^ This same military culture helped to foster a legal culture which protected militarised men accused of sexual misconduct, and this article will argue that Spanish victims of rape often underwent intrusive anatomical examinations with seemingly little benefit to themselves or their legal cases. Through the case study of forensic medicine, this article will therefore underscore the Spanish court system as a site for the secondary victimisation of rape victims under Francoism.

## Forensic medicine and notions of ‘virginity’ under Francoism

Under the Second Spanish Republic of 1931–36 attempts had been made to liberalise certain aspects of Spanish society, which included the legalisation of divorce, the introduction of universal suffrage, and the civic emancipation of women.^11^ The victory of the Francoist side in the Spanish Civil War of 1936–39 saw the consolidation of a traditionalist, military, Catholic dictatorship which reversed the Republic’s liberalising imperatives, banning divorce, discouraging female employment, and subordinating women to the patriarchal authority of the father or husband.^12^ Despite such political upheaval, there appeared to be a good deal of continuity between Francoist legislation relating to sexual crimes and the Republican Penal Code which preceded it. For example, like the 1932 Republican law, the 1944 Francoist Penal Code defined rape as carnal relations with a woman using force or intimidation; when the woman is deprived of reason for whatever cause; or if the woman was under twelve years of age.^13^ In addition, the euphemistic conceptualisation of ‘sexual crimes’ as ‘crimes against decency’ (*delitos contra la honestidad*) reflected understandings of sexual violence which had been dominant for many years.

In 1930s-40s Spain, as elsewhere, rape was in many senses considered a moral crime against what was perceived to be a woman’s most prized asset: her virginity. Regarded as a signifier of a women’s honour, female virginity remained for many a precondition for marriage and thus a source of significant anxiety for women and their families.^14^ In the Spanish context, such understandings of female purity were compounded by the strong influence of Catholicism on courtship culture. Catholic notions of sexual morality persisted within Republican Spain, which espoused Manichean oppositions between ‘good and evil’, ‘sacred and profane’ and ‘unworldly and ‘worldly’.^15^ The advent of Francoist National Catholicism saw a magnification of such ideas, which became official policy under institutions such as the Sección Femenina (Women’s Section of the Falange) and the Patronato de Protección de la Mujer (Board for the Protection of the Woman), the latter established in 1942 to preside over public decency and to police wayward female sexuality.^16^ There was also a ‘bottom-up’ aspect to the policing of female sexuality, and local communities played an important role in fostering an asphyxiating moralising atmosphere in which the fear of scandal and rumour encouraged an important degree of ‘self-policing’ with regards to sexual conduct.^17^ Such social pressures were not unique to Francoism, or even to Spain, but the combination of these more informal forms of social control with the regime’s official policies reinforced traditional restrictions on female sexuality and precluded the possibility of liberalisation which had been glimpsed under the Republic.^18^ With the installation of the dictatorship, the traditional stigma surrounding a woman’s loss of ‘virtue’ also took on an additional, political dimension. In the Spanish post-war, the language of ‘purity’ became bound up with the ‘purification’ of the body politic from the corrupting influence of Republicanism.^19^ As such, long-standing Catholic concerns over female purity combined with Francoist nation building, which incorporated notions of morality and vice within the regime’s process of legitimation.^20^ The legislation relating to sexual crimes was amended to reflect these heightened concerns for female morality. New stipulations in the legislation relating to the sexual crimes of seduction (*estupro*) and corruption of minors (*corrupción de menores*), specified that ‘accredited decency’ (*acreditada honestidad*) now constituted a pre-requisite for legitimate female victimhood.^21^ Though the definition of rape (*violaci­ón*) did not include the ‘decency’ stipulation, court records show that in practice there was a degree of slippage between the different sexual crimes in this regard, and non-virgins, including married women, older women, and mothers, rarely benefitted from this legislation.

Perpetrators of sexual crimes under Francoism were rarely subjected to forensic scrutiny, unless questions had been raised over their mental wellbeing, sexual orientation, or ability to perform penetrative sex. Rather, forensic medical practice relating to sexual crimes centred around the victim, with particular emphasis on examinations of the hymen. The medical examination of rape victims was not necessarily imposed from above. In the case files we can detect a degree of ‘learned process’ in the behaviour of rape victims and their families, whereby some in fact sought out medical examinations, even before reporting sexual crimes to law enforcement.^22^ This in part reflected the desire not to draw public attention to extramarital sexual activity until complainants felt confident of a favourable outcome, and is illustrative of the existence of a general, if imperfect, awareness amongst the Spanish population of the ability of doctors to testify to female sexual experience. More broadly, this acceptance of forensic examinations of the hymen reflected long-standing concerns around female virginity as moral attribute, and as a key marker of female purity and nubility.^23^ But such requests for forensic evidence also testified to the demand for an ‘objective’ arbiter of truth within a highly politicised legal culture heavily reliant on character references (*avales*) from fellow citizens and respected members of the ruling establishment.^24^ This legal tradition worked against those without patrons in the local administration, particularly those with Republican pasts, and so medical evidence was undoubtedly—if erroneously—viewed by some as a corrective to corruption within the courts, particularly when victims were examined by trusted local doctors.

This situation helps to explain the not insignificant number of Republican victims who reported sexual crimes committed by Francoists, even in Francoist-held areas at the height of the Civil War.^25^ In such cases, complainants were sometimes explicit in their requests for medical examinations. In 1939, for example, the mother of a 15-year-old girl who died shortly after an altercation with members of the Civil Guard, requested that her daughter’s body be exhumed and examined for signs of rape.^26^ Beyond the political cleavages of the Civil War, faith in medical evidence could even supplant belief in the testimonies of family members. In a 1937 case in Ceuta, the mother of a sixteen-year-old girl took her daughter to be examined by a doctor before—much to her daughter’s dismay—turning to the authorities to denounce the girl’s boyfriend for sexual assault.^27^ Similarly, in one case from Madrid in 1948, a twenty-year-old man was accused of sexually abusing his eight and ten-year-old cousins while staying in their home. The children’s father stated that he found it difficult to believe that his nephew could have committed such acts, but supported his daughter’s version of events given that a medical examination had revealed minor damage to the hymen.^28^ In contrast, he refused to support the abuse claims of his son, who had not undergone medical examination. In this sense, many believed that medical evidence constituted a robust witness to acts committed in the absence of third-party witnesses.

Forensic medical testimony was, however, far from an objective witness to sexual assault. The hymen has long been acknowledged as a deeply flawed witness both to a woman’s sexual experience and coercive sexual intercourse. A thin layer of tissue covering the vaginal opening, the hymen comes in different forms and can remain intact following sexual intercourse, or be ruptured under circumstances other than penetrative sex. Forensic medical textbooks used by Spanish practitioners acknowledged the ambiguous nature of evidence obtained from studying the hymens of alleged rape victims. For many years, the foremost textbook on forensic medicine in Spain was the Colombian doctor Guillermo Uribe Cualla’s *Medicina Legal y Psiquiatría Forense*, first published in 1934.^29^ Uribe explained that there were different kinds of hymens, which, significantly, included the ‘flexible hymen’ (*himen dilatable*), ‘so elastic that it allows the introduction of a voluminous object without breaking’.^30^ Conversely, Uribe also acknowledged that the hymen could be broken by objects other than a penis, and that in some cases, a severe fever resulting from measles or chicken pox could cause rupture.^31^ Yet while acknowledging the biological ambiguities of the hymen, Uribe simultaneously defined a woman’s ‘deflowering’ in anatomical terms as ‘the rupture of the hymen membrane during coitus practiced with a virginal woman, that is to say, who had an intact membrane.’^32^ Uribe’s mandates were accepted largely uncritically by Spanish practitioners, and later forensic medicine manuals published by Spanish experts show how little scientific expertise on the policing of sex crimes evolved under Francoism.^33^

Spanish professional conferences on forensic medicine under the dictatorship barely mentioned crimes of a sexual nature, and none of the different sections of the Escuela de Medicina Legal (School of Legal Medicine), dealt specifically with such offences.^34^ A 1969 manual written by a collective of Madrid forensic doctors (*médicos forenses*) acknowledged that the hymen could remain intact after coitus while still defining a virgin as a woman who had not had ‘carnal copulation’ and therefore had no damage to the hymen.^35^ This double-speak—in which it was possible to acknowledge the biological ambiguities of the female anatomy while restating the binary doctrine of hymen as signifier of virginity—in many ways reflected contradictions at the heart of Catholic doctrine pertaining to the Virgin Immaculate, whose perpetual virginity allowed for the hymen to survive the birth of Christ intact.^36^ This was, of course, an impossible ideal for the average Spanish female, and such ambiguities therefore helped to foster an uneven state of affairs within the Francoist court room. As Gethin Rees and Ivan Crozier have pointed out, police surgeons were not objective witnesses, but rather helped to reinforce pre-existing social attitudes and beliefs, particularly with regards to gender.^37^ In Francoist Spain, scientific experts not only yielded to prevailing cultural values and popular understandings of female purity, but actively perpetuated rape myths which, as their reliance on Latin American scholarship suggests, were widespread across the western Catholic world.

Most notably, academic forensic doctors helped to reinforce stereotypes relating to the social profiles of the victims and perpetrators of sexual violence. Forensic practitioners were encouraged to adopt a sceptical attitude towards rape victims. It was claimed, for example, that women tended to lie about their sexual histories, and that sexually experienced women would often allege that an assailant had taken their virginity.^38^ In their 1961 textbook on forensic psychiatry, Antonio López Zanón and Antonio Campoy Guerrero echoed this sceptical stance, warning, for example, against placing too much stock in the testimonies of children claiming to have been the victims of sexual assault.^39^ They also stressed the importance of distinguishing victims who were raped while unconscious from ‘the fantasies of hysterical women’ and ‘attempts at blackmail’.^40^ Meanwhile, López and Campoy reinforced broader assumptions of the primitivity and abnormality of sexual criminals. It was stated that these were usually psychopaths or chronic alcoholics, drawn from the lower social classes.^41^ Drawing on Freudian understandings of sexuality as innate but latent in all human beings, they ascribed to the theories of German criminologist Hans von Hentig, notably that the urges of sexual perpetrators could lie dormant until they were activated by ‘the element of seduction’ which emanated from the victim.^42^ As such, the victims of sexual violence were held partly responsible for the abuse they suffered, and were frequently smeared with allusions to prostitution and/or sexual promiscuity in the Francoist courtroom.

The limitations of medical evidence imbued in such rape myths were exacerbated by the difficulties inherent in communicating scientific or medical concepts and terminology within the courtroom. In 1970, forensic doctor Jos­e Maria Reyes Monterreal described the challenges of providing impartial, technical evidence to the courts, particularly when judges expected definitive conclusions on what were merely interpretations, albeit professional ones, of the evidence.^43^ Others called for lawyers and judges to receive training in legal medicine, something which was already compulsory in countries such as Chile, Italy, and Germany but which Spain had thus far failed to implement.^44^ Furthermore, the procedure for medically examining rape victims varied greatly between practitioners. In some cases, for instance, the absence of bruising to the face—where it was assumed that an assailant would hold the victim to prevent her from screaming—could be used to question the victim’s account, while in others, victims who did present facial bruising were disregarded in the concurrent absence of bruising to the genitalia.^45^ This variability was partly due to the infancy of forensic medicine as a professionalised medical specialism. Forensic doctors were only recognised as employees of the state from 1947 and not all jurisdictions had access to trained specialists.^46^

As we shall see, within the courtroom itself, judges were not obliged to defer to forensic evidence, and the courts could, and often did, find ways to omit inconvenient medical testimony. This meant that specialist forensic doctors were under pressure to assert the value of their profession in the courtroom, a struggle for authority which could lead them to overstate the reliability of their findings.^47^ Meanwhile, many victims were assessed by general practitioners, or, particularly if the perpetrator belonged to the armed forces, military doctors, who were not specialists in forensic medicine let alone the specifics of sexual crimes.^48^ As such, the backgrounds of those performing medical examinations on rape victims were varied, and many were unfamiliar with the nuances of the hymen as witness to sexual assault. Nonetheless, given that their medical knowledge of sexual crimes was grounded in assessments of the hymen, forensic doctors were crucial to reinforcing female chastity as a pre-requisite for acceptable victimhood in both the civilian and military courts. The focus on the hymen was thus not driven by the desire to secure justice for victims, but rather promised a straightforward way of rendering sexual deviance legible within a society increasingly concerned with the policing of female sexuality.

## *Forenses* in the courtroom: overstepping the boundaries of medical knowledge

Within each court record there are many unknowns, and we can never know the precise realities of the cases which came before Spanish judges. The testimonies of each party, accuser and accused, were highly mediated, by the public setting of the courtroom, by the presence of family members, and/or by the fear of the consequences that might result from an unfavourable outcome. As such, we cannot read court records relating to rape trials as ‘authentic’ representations of sexual ‘truth’.^49^ Instead, the case files offer an insight into how medical knowledge was mobilised, discussed, and dismissed in the courtroom. Close attention to the medical evidence illuminates the often-subtle ways in which the Francoist courts fostered an environment hostile to the victims of sexual crimes. Forensic doctors were complicit in the courts’ manipulation of medical evidence, often making claims which went beyond the boundaries of contemporary medical knowledge in order to fit the working theory of an investigating judge. This reflected a broader academic medical culture on the Francoist side in the Civil War, which provided a pseudo-scientific gloss to cultural and political prejudices.^50^ Once established, medical experts played an important role in legitimising the Francoist regime, especially on the international stage.^51^ Forensic specialists fed into this broader trend, bringing the apparent legitimacy of science to culturally and politically-contingent understandings of gender and sexual violence. In this sense, far from serving as a corrective to subjective witness statements, medical testimony bolstered a system designed to reinforce patriarchal societal structures by protecting male sexual privilege while holding victims (and their families) responsible for the defence of female purity.

An example of *forenses* stretching the boundaries of medical knowledge to support a particular reading of a case came early on in the Civil War, in August 1936. A thirty-six-year-old civil guard was accused of raping a seventeen-year-old female prisoner in the province of Seville.^52^ The victim maintained that she had been a virgin prior to the attack, and pointed to a bruise on her left arm as evidence of use of force. The two forensic doctors presiding over the case stated that the victim’s body showed no signs of violence, neither on her thighs or on her external genitalia. They also cast doubt on the bruise on her arm, stating that if she had been grabbed, the marks of the other fingers would have been present. Their internal examination revealed no marks or inflammation on the vulva or vagina, but noted the absence of the hymen, and though they were unable to confirm when the ‘defloration’ had occurred, they stated that if the victim had been raped when she claimed, the tears would have been visible. This assertion failed to take into account the heterogeneity of hymens, and no appreciation was given to the seven-day delay between the incident and the medical examination. Yet the forensic medical report was instrumental in dismissing the case against the perpetrator, and the charge was deemed ‘absolutely false’ by the presiding judge.

Another striking example of forensic overstretch came in a 1942 case in Ceuta, an ethnically diverse Spanish enclave located in northern Morocco just across from the straits of Gibraltar.^53^ The parents of a fourteen-year-old girl alleged that she had been raped by a thirty-six-year-old chauffeur of ‘Moorish’ heritage while working as a maid in his house. The girl recounted that her employer, for whom she had been working for the past three years, lured her into the garage where he expressed his desire to marry her. At this point, he pulled her into his car where he ‘abused and dishonoured her’. Despite the significant age gap, the perpetrator presented the relationship as consensual, confirming that their first sexual contact had been in his car, and adding that the pair had ‘cohabited’ various times, always with the girl’s consent. Though the mother attempted to mobilise her racial privilege, drawing on derogatory racialised language such as ‘indigenous’ and ‘Moorish’ to underscore the perpetrator’s deviance, this paled in comparison to the man’s relative class privilege, reflected in his status as a property and car owner, well integrated into the networks of the local professional classes.^54^

The court was keen to accept the perpetrator’s version of events. During a face-to-face confrontation (*careo*), a court official reported how the accused spoke ‘affectionately’ and ‘with gentleness’ to the girl, whose purported irrationality was betrayed by the ‘shame’, ‘energy’ and ‘violence’ with which she disputed his claims.^55^ The forensic doctor and general practitioner charged with carrying out the forensic examination bolstered the perpetrator’s narrative. The medical examination, which was carried out some four months after the alleged attack and included the highly dubious ‘two-finger’ test, asserted that the girl’s hymen was intact apart from two small slits which were either congenital or scarred over from tearing. They added that the hymen was flexible enough ‘that it has been possible to repeat the acts of coitus without leaving a trace beyond what has been described’. Implicit in this claim were the assumptions that first, the girl was not a virgin, and second, that sex had taken place multiple times. The latter again prioritised the perpetrator’s narrative over the girl’s insistence that coitus had only taken place once, the perpetrator having assured her that they would not have to do it again until they married. These subjective judgements shaped the doctors’ supposedly scientific conclusion that ‘the most feasible is that coitus was consensual (*permitido*), given that if it had not been there would have been greater tearing, although given the aforementioned anatomical constitution, in exceptional circumstances this tearing might not have occurred.’ The consent narrative promoted by both the perpetrator and those involved in examining the evidence took no account of the age difference or power wielded over the girl by her long-term employer. What counted on this occasion was the perpetrator’s status, as well as his stated desire to marry the girl, which mitigated the crime in the eyes of the authorities despite protests from the girl’s parents. Within this context, the medical witnesses were happy to validate the assumptions of the investigating judge.

## The exploitation of medical ambiguity by court officials

An examination of the court records reveals that judges and other court officials were well aware of the ambiguities of medical evidence, and could set the parameters of an examination in order to shape its outcome. Spain’s discretionary court system also ensured that judges presiding over rape cases could pick and choose which elements of one or multiple medical testimonies best fit with their perception of the perpetrator’s guilt, a perception in turn shaped by rape myths or even a sense of military brotherhood or personal affinity with the accused. Most commonly, investigating judges could direct certain leading questions to doctors which would inevitably draw attention to the weaknesses of the medical evidence. This could be an effective strategy in even the most seemingly clear-cut of cases. In early 1940, a twenty-eight-year-old married civil guard was accused of raping a thirteen-year-old girl in the province of Cádiz, the daughter of the commanding sergeant of the station where the guard worked.^56^ According to the victim, on 5 February the assailant had lured her into a neighbour’s chicken coop under the premise that one of her hens had wandered inside. He then attempted to rape her but was interrupted in the act by the girl’s mother. The girl in question subsequently revealed that the accused had already raped her on a secluded path on 21 January when she was out buying bread. An initial medical examination, carried out on 6 February 1940 by two general practitioners, noted the rupture of the hymen, though added that they ‘considered that the introduction of the penis into the vagina would have been difficult given its observed narrowness’. This interpretative gloss—which in fact contradicted medical textbooks stating that it was entirely possible for a grown man to rape a pre-pubescent girl—made space for the subsequent undermining of the victim’s narrative.^57^

On 15 February 1940, the doctors who had compiled the original forensic report were asked to answer some follow-up questions, specifically if the hymen had been ruptured by a penis, or any other object. This was an unusually direct question, and one in any case, in the absence of semen—inevitable given the time lapse between the assault and examination—it was impossible for them to answer definitively. Unsurprisingly, therefore, in answer to this question, one doctor responded that the wound was ‘not recent’ and that it could have been caused by the penis or ‘any other circumstance’. The other stated that the ‘deflowering’ dated from more than ten days and that this could have been caused by a penis, another object, or ‘a simple fall’. The fact that the ‘deflowering’ was dated to over ten days was in fact in line with the victim’s timeline of events. Nonetheless, the tone of proceedings subsequently changed, with greater emphasis being placed on the ambiguity of the medical testimony. On 30 August, a statement made by the public prosecutor (*fiscal*) drew attention to the legal definitions of rape, which for girls over the age of twelve specified the use of violence or intimidation, or the lack of consciousness of the victim. In this case, the *fiscal* emphasised that there was no evidence that force had been used, adding that:

Once the girl [name] was examined by the doctors, these certified that she had been deflowered given the rupture of the hymen, though they considered that it would have been difficult to introduce the penis into the vagina given the narrowness of the latter, that the rupture was not recent and that it could just as well have been caused by the penis as any other object or from a simple fall, [and] that it dates from more than ten days … 

The *fiscal* reiterated this stance on 30 December 1940, emphasising the absence of violence and stating that there was no definitive confirmation that coitus had taken place at all. The final judgement absolving the accused on 11 February 1942 once more referenced the forensic report, now presenting this as ‘proven fact’ (*hechos probados*):

That the minor in question according to the medical report is deflowered given that the hymen is ruptured, though the doctors in question consider it would have been difficult to introduce the penis into the vagina, given the narrowness of the latter and that the rupture is not recent and that it could have been caused by the penis, by any other object, or by a simple fall, That the accused is not a minor and has no previous convictions. FACTS THAT ARE DECLARED PROVEN.

The exact phrasing has been included here in order to convey the process by which ambiguous and even inaccurate medical testimony could be absorbed into the bureaucratic language of the courts and repeated over the course of a long trial process until it became incontestable fact. Though absolved of rape, the court indicated that the accused could be prosecuted for the lesser crime of seduction (*estupro*), the charge used to prosecute non-violent sexual intercourse with virgins between the ages of twelve and twenty-three committed using ‘trickery’. There is no evidence that this option was ever pursued by the victim and her family. Having been embroiled in a legal case which had now lasted two years, and having witnessed the ease with which seemingly concrete medical and testimonial evidence could be dismissed, it is likely that they deemed it best to put an end to the ordeal. The medical testimony which cast doubt over the anatomical possibility of rape drew on the more general tendency to disbelieve the testimonies of children in favour of perpetrators in positions of authority, and the questions subsequently put to the doctors about the nature of the hymen’s rupture further highlights both the ambiguity of medical evidence in sexual violence trials, and the ease with which potentially damning evidence could be rendered utterly ineffective.

While reflecting more generalised rape myths, interpretations of medical evidence were also sensitive to Spain’s political turmoil, particularly during the Civil War and in the early post-war years. Following the Francoist victory in a particular area during the war, the new authorities carried out the extensive repression of those who had supported the Republic. This included extrajudicial killings, economic pillaging of the vanquished, as well as the purging of the public sector.^58^ Incidences of sexual violence during this period often reflected this repressive context, when officials, militias, or opportunistic assailants were able to exploit their authority or the general disruption of civil war with impunity.^59^ This was particularly the case in the late 1930s and early 1940s, and medical evidence was easily manipulated in order to protect the military and political allies of court officials. An example of this can be seen in a case dating from 1937 in Ronda (Málaga), where two nineteen-year-old cousins accused three Falangist militiamen of raping them after being forced into their car at gunpoint and threatened with castor oil and head shaving.^60^ Rather than deny that sexual relations had occurred, the defendants claimed that the women had consented to sexual relations. The medical report for both women went beyond the limits of medical knowledge to support this defence, and played an important role in discrediting their testimonies. Identical forensic reports written nearly two weeks after the incident certified that there were no signs of recent rape, but that both women were ‘deflowered’ ‘probably from engaging in intercourse more than once’. In the judge’s summary, the qualification was dropped, and the probable frequency of their sexual encounters became a matter of fact: ‘they present deflowering after having engaged in intercourse more than once’. In this sense, despite its shaky basis in scientific fact, the forensic report offered apparently conclusive evidence of the victims’ negligence and dishonour. Under the weight of mounting evidence against them, the fathers of both girls ‘pardoned’ the assailants, which brought an end to the criminal proceedings.^61^

The focus of forensic doctors on the hymen as evidence of sexual violence was problematic for victims who were not virgins prior to the assault, including women whose sexual relations were legitimated by wedlock.^62^ There was a political dimension to this; the emphasis on virginity discriminated against women who had embraced the liberalising agenda of the Republic and were cohabiting with partners to whom they were not married. This was the case for the victims of a rebel militiaman and serial rapist operating in the Loja area (Granada) in 1936, who abused four women in their homes on separate occasions, taking advantage of the absence of their Republican menfolk.^63^ In this case, the medical evidence disregarded the first two victims, given that one was already living a ‘marital life’ (*vida marital*) with her long-term partner, while the other purportedly showed no signs of rape given that she had already given birth several times. The allegations were taken seriously nonetheless, particularly as further victims came forward and a number of unfavourable references emerged from both the perpetrator’s military colleagues and the local townhall. Such testimonial evidence ensured that, despite his allegiances to the rebel side, the accused was found guilty and condemned to thirty years in prison.

It is important not to overstate the centrality of Spain’s political cleavages to the policing of sexual violence throughout the Francoist dictatorship. While rape constituted one aspect of gendered political violence during the conflict, and could also be perpetrated by opportunistic individuals taking advantage of the prevailing upheaval, there were many other ‘constellations’ of sexual assault reported under Francoism beyond the bipolar Civil War logic of ‘blue’ rapes of ‘red’ women (and vice-versa).^64^ Numerous cases can be found in the court records in which perpetrators were absolved despite leftist leanings that might have incriminated them, and conversely, as the Loja case shows, political adherence to Francoism was insufficient to protect assailants from a guilty verdict in the face of overwhelming witness testimony.^65^ In this sense, the handling of rape victims by the courts was less demarcated by the dictatorship’s binary victor-vanquished narrative of the Civil War than by the more generalised retrenchment of conservative, Catholic values within Spain. Victims of sexual violence who came before the courts were thus less likely to have been abused by strangers in politically-motivated attacks than by those who lived or worked in close proximity to them. Young women and girls from low-income families who worked as domestic labourers were particularly at risk. Working alone, often over long periods of time in the company of men much their senior, maids and nannies were vulnerable to sexual abuse which was subsequently difficult to prove. In such cases, medical assessments were frequently shaped by class-based prejudices, which held the victims to be partially, if not wholly, responsible for their abuse.

In 1949, a thirty-three-year-old artillery sergeant was accused of raping a twelve-year-old girl working as a nanny in his home in Cordoba.^66^ Sexual intercourse with girls under the age of twelve was deemed rape even without the use of force or intimidation, and therefore the outcome of the case rested upon proving that coitus had occurred between both parties. An initial forensic report by a local general practitioner testified that the girl’s hymen had a ‘small slit approximately one millimetre deep, akin in form to a small scarred-over tear, in the top right, similar to what could be caused by an attempt to insert a penis or other object of similar shape and dimensions’. In addition, the girl tested positive for gonorrhoeal vulvovaginitis, an assessment which seemed to offer conclusive evidence of the transmission of venereal disease during sexual abuse. Nonetheless, the court subsequently ordered for the girl to be examined by a specialist forensic doctor to determine if she had been ‘deflowered’, when this had occurred, and if she was pregnant. In contrast to the brevity of most medical certificates, this second report spanned three pages, and constituted a detailed discussion of the ambiguous nature of gonorrhoeal vulvitis in young girls. The *forense* explained that vulvitis was very common in girls, ‘especially in those with poor hygiene, to the extent that legal doctors such as Coutagne state that vulvitis is normal in girls who belong to inferior social environments’.^67^ The report also raised doubts over the venereal origins of gonorrhoea, stating that this infection could be transmitted in a number of ways not limited to sexual intercourse, ‘although it is in many cases’. The forensic doctor concluded that in any case, the girl currently presented no symptoms of infection, had not been ‘deflowered’, and that any vaginal discharge would need to be analysed under a microscope by a specialist in order to determine its origin.

This report was revealing of the role of forensic doctors, and particularly of the ‘boundary work’ being performed in the courtroom during this period.^68^ Without specialist equipment, the forensic doctor was in no better position than the general practitioner to speak to the presence of venereal disease. Yet his deployment of the specialist theory granted him authority over the first medical testimony. Despite offering no additional evidence, the forensic doctor introduced apparently scientific victim-sceptic narratives into the courtroom, particularly that young girls with vaginal infections were prone to inventing malicious accusations.^69^ Despite its flawed foundations, the apparent specialist nature of the report was enough to override the previous medical certificate, and given that it was now deemed that the girl had not been ‘deflowered’, the civilian court stipulated that the perpetrator could not be tried for *estupro*, and that a prosecution of attempted rape (*tentativa de violación*) or dishonest abuse (*abusos deshonestos*) constituted the jurisdiction of the military courts.^70^

Once passed over to the military court, the girl was subjected to a third medical examination, now carried out by three military doctors over two months after the incident. This examination certified that the girl was suffering from eczema around her vulva, almost certainly caused by the purulent discharge coming from the vagina. They added that the hymen appeared torn with the remains in the process of scarring. From this assessment they concluded that the girl had been deflowered, and that this had occurred forty to sixty days prior ‘following the introduction of an erect human penis or an instrument similar in shape and size’. Though this finding conformed to the victim’s version of events, they could not determine whether the girl’s infection was of venereal origin as they claimed they lacked the facilities to determine this. Yet conversely, a medical examination of the perpetrator certified that ‘he does not currently have any syphilitic-venereal disease’ (*no presenta en la actualidad enfermedad venereo-sifilítica alguna*). It is unclear how the medical tribunal (comprised of nearly the same team of doctors) was able to prove that the perpetrator was not suffering from venereal disease, while at the same time being unable to test the girl for the same infection. In addition, no consideration was given to the fact that two months had passed since the attack, during which time the perpetrator might have been treated for disease. Similarly, no further tests were ordered on the victim’s sample to establish a definitive diagnosis of her condition. Most significantly, in a court summary of the reports, the ‘syphilitic’ qualification was dropped, and the perpetrator was said not to present ‘any kind of venereal disease’ (*no presenta enfermedad venérea alguna*). Given that the girl had originally been diagnosed with a gonorrhoeal infection, the statement pertaining to syphilis was irrelevant, but did grant the courts an (albeit false) basis upon which to dismiss the otherwise robust evidence that the transmission of venereal disease had occurred. This almost imperceptible bureaucratic smudging enabled the court to dismiss the case, citing the absence of witnesses and the perpetrator’s negative venereal disease diagnosis.

As the allusion to the victim’s class in the 1949 case suggests, the perceived ‘decency’ of the victim was fundamental to the development of a rape case and could serve to undermine apparently robust medical evidence. In a 1950 rape case in Zaragoza, the twenty-nine-year-old victim claimed that she had just left her boyfriend’s home in the early hours of the morning when she was attacked by a stranger.^71^ The man had offered to take her to the nearest tram stop but instead took her to an isolated spot where he raped her. She went to her local community health centre to be examined almost immediately after the attack, where the on-call doctor certified that she showed signs of a previous pregnancy, as well as apparently recent lesions on her labia majora and vagina which were indicative of rape. Yet the police officers to whom the victim reported the case soon raised suspicions over the woman’s testimony. It transpired that she had already had two pregnancies out of wedlock to two different men, and her father made a damning statement in which he explained that he had chucked her out of the family home upon learning of the second pregnancy. He added that he did not think his daughter was ‘normal’, ‘whether that might be due to the nerves or something else’.^72^ The perpetrator, a thirty-four-year-old air force chauffeur, compounded the wayward image of the victim, stating that the woman had proposed sexual intercourse as she did not have anywhere to stay, and that he had paid her ten pesetas in exchange for the act. Revealing in this file was the refusal of those investigating the case to use the term ‘rape’, despite the appearance of this word in the medical certificate. The front page of the court file, for example, referred to the incident as ‘the facts attributed to the driver [name] by Miss [name]’. In his final remarks, the investigating judge underscored the victim’s ‘dubious morality’, and concluded that no crime had been committed. No reference was made to the medical certificate: regardless of the initial medical report, implicit throughout the investigation was the understanding that certain (indecent) women could not be raped.

Conversely, women who were proven ‘virgins’ on the basis of medical testimony could also find it difficult to find justice. On Saturday 8 November 1947, an eighteen-year-old woman was taking an afternoon walk near her home in Madrid when she was approached by a thirty-four-year-old captain of the air force.^73^ The man invited the woman to accompany him on a walk, and the couple spent the rest of the day drinking in various establishments until at three am, inebriated, they went back to the man’s apartment. The following day, the girl told inspectors at her local police station in Madrid that the man had locked her in the flat against her will and, while she was immobilised from the drink, proceeded to rape her. The judge presiding over the case called for a medical examination to determine whether the plaintiff had been ‘deflowered’, and whether this ‘deflowering’ was recent and showed signs of violence. The examination noted a congenital groove and a large central opening in her hymen, adding that the latter demonstrated ‘great elasticity’. Notwithstanding these comments, the forensic doctor carrying out the examination stated that the woman showed no traumatic lesions, concluding that ‘she has not been deflowered, that is, she is a virgin’.

This judgement proved crucial to the development of the case. Though she maintained that she had awoken in the man’s flat to find her skirts raised, experiencing pain to her genitals, the woman was unable to confirm that she had witnessed the act given her lack of consciousness. Her lawyer attempted to contest the medical evidence, drawing attention to the doctor’s remarks on the elasticity of her hymen, which made it possible for sexual intercourse to have occurred without rupture. Yet now that the forensic doctors had seemingly assured the woman’s chastity, there could be no rape charge and the matter was passed over to the military court which, given the man’s profession, was deemed the competent authority. Like many such cases, the legal process advanced very slowly, and it took until May 1948 for the case even to be passed over to the new ruling authority. At this time, the woman was subjected to a further, more intrusive, round of questioning, which probed into her family’s living arrangements and income. Soon after, the general auditor reported that the medical report had ‘accredited’ the woman’s virginity, and that her family were withdrawing their complaint. This case points to the flipside of the hymen’s centrality within criminal proceedings relating to rape. As the embodiment of sexual purity, the hymen constituted the ultimate signifier of justice/injustice within rape trials. The policing of sexual crimes under Francoism was not driven by a desire to recognise female pain or bring violent criminals to justice—it is worth noting here that ideas of ‘rape trauma’ would not be developed by international psychiatrists until the 1970s—, but rather to offer redress for the spoiling of otherwise nubile women.^74^ In this context, the woman’s physical or psychological pain was inconsequential; with her chastity apparently intact, there was no crime.

The conflation of chastity with the physicality of the hymen became problematic on occasions where the courts were forced to confront the limitations of this doctrine. In a 1941 case, one woman accused a thirty-five-year-old man of the rape by means of trickery (estupro) of her fifteen-year-old daughter.^75^ As in many such cases, the man had hidden his marital status and encouraged the girl under the promise of marriage to engage in illicit relations with him, on this occasion in the anteroom of a gallery seat at a nearby cinema.^76^ What was unusual about this case was that the girl had fallen pregnant, but both parties agreed that they had not engaged in full sexual intercourse. Three medical examinations confirmed this narrative: anatomically-speaking, the girl was a pregnant ‘virgin’.

This situation created a confusing state of affairs. Under ordinary circumstances, the confirmation of the girl’s ‘honour’ would have been sufficient grounds to dismiss the *estupro* charge. Yet the pregnancy constituted incontestable evidence of carnal relations, and the mother and daughter were keen to secure financial support for the child. Perhaps remarkably given the medical ambiguities of the case, no specialist *forense* was called to offer an expert opinion, and therefore no medical detail was provided over the potential elasticity of the girl’s hymen, which might have permitted sexual intercourse without rupture. As it was, there was some confusion over whether the *estupro* charge could be upheld if, despite the pregnancy, the girl had not been deflowered. Initially proceedings seemed to be heading in favour of the plaintiff. The girl had excellent character references from those who knew her, and the perpetrator had seemingly incriminated himself by paying 1500 pesetas towards the upkeep of the pregnant mother and child. In addition, seemingly acting upon the advice of a midwife—who according to different versions of the story had told the man the girl would need to be ‘deflowered’ if they wanted to either provoke a miscarriage or facilitate the eventual birth—the man proceeded to consummate the relationship, which the girl claimed had been against her will and using force. A subsequent examination indeed confirmed that the girl had now been ‘deflowered’, potentially adding the more serious rape charge to the initial *estupro* denunciation.

Yet no rape prosecution was pursued. Instead, a concerted smear campaign was launched against the girl’s mother in a bid to clear the perpetrator of all charges. The man accused the mother of attempting to ‘sell’ her daughter and, more drastically, denounced her for attempted blackmail. The mother was described as a former cabaret dancer, who had tried to convince the man to engage in commercial sex with her daughter. When he refused, the mother was accused of insinuating to others in the community that he was having sex with her daughter in a bid to blackmail him. The perpetrator found a number of sympathetic witnesses (mainly friends of his) to corroborate his story. Significantly, this included a new statement from one of the aforementioned doctors—also an acquaintance of the accused—which reiterated that when he had examined the girl some months prior, she retained ‘the physical signs of virginity’ despite the pregnancy, and explaining that her ‘mother who had been a prostitute wanted to check that she was a virgin’. By this point in proceedings, the original *estupro* charge, and indeed the many inconsistencies in the perpetrator’s account, were dwarfed by the new spiral of accusations against the mother. In the face of such intimidation, the mother withdrew the complaint, ‘pardoning’ the accused of all wrongdoing and thereby absolving him of responsibility for the child. In many ways, the fifteen-year-old girl at the heart of this case was the ‘perfect’ victim; like the Virgin Mary herself, she had somewhat miraculously achieved conception while maintaining the physical signs of ‘virginity’.^77^ In other words, despite her assault she remained ‘honourable’. It is indeed noteworthy that the perpetrator chose not to focus on the girl’s honour in his attempts to dismantle the case. Faced with the charge of assaulting an ‘accredited’ virgin, the best strategy was to focus on discrediting the mother. In this sense, even when they conformed to its tenets, the doctrine of the hymen-as-signifier-of-purity was ultimately insufficient to protect young victims of sexual violence from experiencing a ‘second rape’ within Spain’s corruptible court system. Inconvenient medical testimony was easily rendered insignificant under the weight of overwhelming testimonial evidence.

## Conclusion

Medical evidence offered victims hope that their complaints would be taken seriously, regardless of their background or reputation. This was particularly important within the context of Spain’s Civil War and aftermath, during which time the legal system had been heavily exploited in order to repress the nascent Francoist regime’s political enemies. However, while court officials welcomed the aura of objectivity that medical evidence offered, they frequently exploited its ambiguities in order to support their working theories. The emphasis on the hymen offered the courts a seemingly clear-cut way to address the thorny issue of sexual abuse. If intact, an accusation of rape could be easily dismissed. Meanwhile, a ruptured hymen might indicate sexual activity, but said little about the circumstances of this. The discussion then shifted to an assessment of both parties’ relative credibility, one which largely depended on testimonial evidence and was often decided in the perpetrator’s favour. In many cases, complainants were so intimidated by this process that they opted to drop the charges altogether. The presence of forensic evidence did little to mitigate the manipulation of evidence, and *forenses* were often only too willing to corroborate the steer of the court. This willingness resulted from a professional training steeped in rape myths, combined with the pressure to demonstrate the merits of their budding profession, and the influence of their own personal prejudices. Though held in high esteem, medical evidence came second to testimonial assessments of the victims and perpetrators.

The emphasis on the hymen within forensic medical assessments reflected broader Francoist understandings of sexual violence, which was not regarded as a traumatic, violent assault on the individual, but rather as a crime against ‘decency’ and, implicitly, an attack against women and girls as male property. The vast majority of cases investigated by the courts involved young, female victims, many of them children—in other words, virgins who were perceived to have the most to lose from sexual violence in terms of future eligibility for marriage. Such understandings were not necessarily specific to Francoist Spain. As the influence of Latin American and other European scholarship on the work of Spanish *forenses* shows, such patriarchal understandings of female sexuality present in the Francoist courtroom were also embedded in international theory on sexual crimes. In this sense, the limitations of the hymen as witness under Francoism were in part due to the contradictory scientific knowledge available to forensic doctors at the time, which combined anatomical and more generalised culture-based assessments of the female body. Yet, as such contradictory discussions of the hymen within contemporary medical forensic textbooks attest, medical evidence was *always* ambiguous; what stood out in the Francoist case was the degree of discretion available to court officials to render this ambiguity visible within the legal process. The ambiguities of medical evidence were thus opportune within Francoism’s paternalistic, politically-repressive, and unequal society, as it enabled those in positions of power to protect their own interests while reinforcing patriarchal societal structures. Forensic doctors were therefore active participants in the second victimisation of rape victims during this time. Forensic medicine served as an ally to Francoist paternalism, as its ambiguous testimony could be exploited while projecting an image of absolute truth.

## Notes

1 See, for example, Carme Molinero, ‘Mujer, franquismo, fascismo. La clausura en “un mundo pequeño”’, *Historia Social* 30 (1998): 97-117; Pilar Folguera, ‘La construcción de lo cotidiano durante los primeros años del franquismo’, *Ayer* 19 (1995): 165-187; Rosario Ruiz Franco, ‘Legitimar a golpe de ley: la imposición de la mujer ideal franquista’ in *La Alargada Sombra del Franquismo: Naturaleza, Mecanismos de Pervivencia y Huellas de la Dictadura*, ed. Asunción, Dunia Etura, and Mattero Tomasoni (Granada: Comares, 2019), 231-248; Aurora Morcillo, ‘Shaping True Catholic Womanhood: Francoist Educational Discourse on Women’, in *Constructing Spanish Womanhood: Female Identity in Modern Spain*, ed. Victoria Loree Enders and Pamela Beth Radcliffe (Albany: State University of New York Press, 1998), 51-69.

2 On these debates see Pura Sánchez, *Individuas de Dudosa Moral: La Represión de las Mujeres en Andalucía (1936-1958)* (Barcelona: Crítica, 2009), 215-231; Maud Joly, ‘Las violencias sexuadas de la Guerra Civil Española: paradigma para una lectura cultural del conflicto’, *Historia Social* 61 (2008): 89-107; Adriana Cases Sola, ‘La violencia sexual en la retaguardia Republicana durante la Guerra Civil Española’, *Historia Actual Online* 34 (2014), 69-80; Laura Muñoz Encinar, ‘Unearthing gendered repression: an analysis of the violence suffered by women during the Civil War and Franco’s dictatorship in Southwestern Spain’, *World Archaeology*, 51, no. 5 (2019): 759-777; Ángel Alcalde, ‘Wartime and post-war rape in Franco’s Spain’, *The Historical Journal*, 64, no. 4 (2021): 1060-1082; Ángel Alcalde, ‘Queipo de Llano, Koestler y las violaciones de mujeres en la guerra civil española’ in *Mujeres, Género y Violencia en la Guerra Civil y la Dictadura de Franco*, ed. Conxita Mir and Ángela Cenarro (Valencia: Tirant humanidades, 2021), 81-116; Noemí Riudor i Garcia, ‘Violaciones durante la Guerra Civil: Ecos de una realidad’ in Mir and Cenarro, *Mujeres, Género y Violencia*; 215-243.

3 On the idea of the ‘second rape’, see Lee Madigan and Nancy C. Gamble, *The Second Rape: Society’s Continued Betrayal of the Victim* (New York: Lexington Books, 1991); Rebecca Campbell et. al, ‘Community Services for Rape Survivors: Enhancing Psychological Well-Being or Increasing Trauma?’, *Journal of Consulting and Clinical Psychology* 67, no. 6 (1999): 847-858; Patricia Yancey Martin and Marlene R. Powell, ‘Accounting for the “Second Assault”: Legal Organizations’ Framing of Rape Victims’, *Law and Social Inquiry* 19, no. 4 (1994): 853-890.

4 See, for example, Georges Vigarello, *A History of Rape: Sexual Violence in France from the 16th to the 20th Century* (Cambridge: Polity, 2001), 39-40; Joanna Bourke, *Rape: A History from 1860 to the Present* (London: Virago, 2013), 22-24.

5 Joanna Bourke, ‘Police Surgeons and Victims of Rape: Cultures of Harm and Care’, *Social History of Medicine* 31, no. 4 (2018): 720-24.

6 Juan Manuel Jiménez Muñoz, *Historia legislativa del cuerpo de médicos forenses* (Universidad de Valladolid: Valladolid, 1974).

7 See, in particular, Rose Corrigan, *Up against a wall. Rape reform and the failure of success* (New York: NYU Press, 2013).

8 *Boletín Oficial de Estado* (henceforth BOE), 204, 23 July 1945, ‘Código de Justicia Militar’, art. 13.

9 Gabriel Cardona, *El gigante descalzo: El ejército de Franco* (Madrid: Aguilar, 2003), 97, 105. Military historian Gabriel Cardona estimates that in the early 1940s, the Spanish army comprised between 400,000 and 750,000 troops. On the protagonism of military justice under Francoism see, for example, Antonio Andrés Laso, ‘Legislación penal, procesal penal y penitenciaria tras la Guerra Civil Española’, *Derecho Penal* 35 (2015): 12, 18; Peter Anderson, *The Francoist Military Trials: Terror and Complicity, 1939–1945* (New York: Routledge, 2010)*.*

10 Alcalde, ‘Wartime and post-war rape in Franco’s Spain’, esp. 1065, 1072.

11 Ruiz Franco, ‘Legitimar a golpe de ley’, 231-248.

12 Mary Vincent, *Spain 1833-2002: People and State* (Oxford, 2007), 169; Rosario Ruiz Franco, ‘La Situación Legal’, in Gloria Nielfa Cristóbal (ed.), *Mujeres y Hombres en la España Franquista: Sociedad, Economía, Política, Cultura* (Madrid, 2003), 122; *BOE*, 199, ‘Fuero de los Españoles’, 18 July 1945. See most notably the reinstatement of the 1889 Civil Code, which criminalised abortion, contraception, and divorce. Similarly, the 1939 ‘Labour Charter’ (*Fuero de Trabajo*), defined the family as ‘the moral institution endowed with inalienable rights and superior to all positive law’.

13 (BOE), Código Penal, 13 January 1945. This definition left ample space for discretion in defining exactly what constituted ‘force or intimidation’ and ‘deprived of reason’, and was common to other contexts. See, for example, Joanna Bourke, ‘Sexual Violence, Bodily Pain, and Trauma: A History’, *Theory, Culture and Society* 29, no. 3 (2012): 33-35.

14 On virginity as moral attribute see Vigarello, *History of Rape*, 14, 29-30; Efi Avdela, Kostis Gotsinas, Despo Kritsotaki and Dimitra Vassiliadou, ‘From virginity to orgasm: marriage and sexuality in twentieth-century Greece’, *Journal of Family History* 45, no. 3 (2020): 320; Laura M. Carpenter, *Virginity lost: An intimate portrait of first sexual experiences* (New York: New York University Press, 2005), 23.

15 Mary Vincent, ‘Gender and morals in Spanish Catholic youth culture: A case study of the Marian congregations’, *Gender and History* 13, no. 2 (2001): 276.

16 Aurora Morcillo, ‘Walls of flesh. Spanish postwar reconstruction and public morality’, *Bulletin of Spanish Studies* 84, no. 6 (2007): 743. One of the Patronato’s main responsibilities was to keep watch on young women, incarcerating those deemed promiscuous or ‘at risk’ of falling into disrepute. On the Sección Femenina, see Inmaculada Blasco Herranz, *Armas Femeninas para la Contrarrevolución: La Sección Femenina en Aragón (1936-1950)* (Malaga: Servicio de Publicaciones y Divulgación Científica de la Universidad de Málaga, 1999).

17 See Gloria Rom­án Ruiz, ‘¿El baile agarrado es pecado? La burla del control social en los espacios de “inmoralidad” del campo alto-andaluz durante el franquismo’, *Cuadernos de Historia Contemporánea* 42 (2020): 324.

18 The work of the poet and playwright Federico García Lorca (1898-1936) constitutes the most iconic depiction of the suffocating role of the community in pre-Civil War Spain, particularly with regards to questions of family honour and unrequited romantic desire. See, for example, Lorca’s ‘rural trilogy’, which includes *Bodas de Sangre* (1932), *Yerma* (1934), and *La Casa de Bernarda Alba* (1936).

19 Destitute Republican women pushed into prostitution were particularly vulnerable to the Patronato’s moralising imperatives. Morcillo, ‘Walls of Flesh’, 110; Sánchez, *Individuas de dudosa moral*; Michael Richards, ‘Morality and biology in the Spanish Civil War. Psychiatrists, Revolution and Women Prisoners in Málaga’, *Contemporary European History* 10, no. 3 (2001): 395-421. For more on ‘purification’ of the regime’s enemies, see Michael Richards, *A time of silence: civil war and the culture of repression in Franco’s Spain, 1936–1945* (Cambridge: Cambridge University Press, 1998).

20 Richard Cleminson and Claudio Hernández Burgos, ‘The purification of vice: early Francoism, moral crusade, and the *barrios* of Granada, 1936-1951’, *Journal of Spanish Cultural Studies* 16, no. 1 (2015): 96.

21 BOE, Código Penal, 13 January 1945, art. 434. In Francoist Spain *estupro* was a broad term which referred to various kinds of illicit sexual intercourse, including incest; carnal relations out of wedlock with a virginal woman between 12 and 23 years old involving an abuse of power; and sex with a woman between the ages of 16 and 23 involving ‘trickery’ (*engaño*). The *estupro* charge was often used by women claiming to have slept with their boyfriends following a promise of marriage which never materialised.

22 Karen Dubinsky, ‘“Maidenly Girls” or “Designing Women”? The crime of seduction in turn-of-the-century Ontario’, in *Gender conflicts: New essays in women’s history*, ed. Franca Iacovetta and Mariana Valverde (Toronto: University of Toronto Press, 1992), 49. Dubinsky describes how in turn-of-the-century Canadian ‘seduction’ trials, the very fact of calling the police, consulting a lawyer, and attending court were ‘learned processes’ which citizens had learnt to follow over time. For some examples in the Spanish context see Archivo Histórico del Ejército del Aire (henceforth AHEA) C.8540, C.17705, C.11257, C.18357; Archivo General de Ceuta, Fondo: Archivo Judicial de Ceuta, (henceforth AGC), Caja 143, exp. 2.

23 Vigarello, *History of Rape*, 14, 29-30; Avdela, et. al., ‘From virginity to orgasm’, 320; Carpenter, *Virginity lost*, 23.

24 See, for example, Anderson, *Francoist Military Trials*.

25 Some examples include Archivo Histórico del Tribunal Militar Territorial Segundo de Sevilla (henceforth TMTS) 8000, leg. 105, no. 2965; Causas, leg. 109, no. 3122; 8000, 134, 4724; 8000, leg 112, no, 3286; Causas, leg. 184, no. 8136; Causas, leg 104, no. 1869; Causas, Leg. 462, no. 6831.

26 TMTS, Causas, Leg. 160, 2086.

27 AGC, Caja 143, exp. 2.

28 AHEA, C.13637.

29 Guillermo Uribe Cualla, *Medicina Legal y Psiquiatría Forense* (Madrid and Bogotá, 7th edn: Ediciones Guadarrama, S. L., 1957), 400. Uribe’s work was eventually printed in Spain in 1957.

30 *Ibid.*, 403,406.

31 *Ibid.*, 406.

32 *Ibid.*, 403.

33 See, for example, Antonio López Zan­ón and Antonio Campoy Guerrero, *Psiquiatría Forense: Tomo I* (Madrid, 1961).

34 Ángel de Alba y Osuna and Antonio Sabater Sanz, ‘El Servicio Médico Forense en España. El Informe Médico Forense en el Juicio Oral’, *Anales de Medicina Forense de la Asociación Española de Médicos Forenses* (1968-1972)**,** 327.

35 Vicente Moya Pueyo, Manuel Sancho Lobo, José A. García Adrade and Fabriciano Jiménez Cubero, *Medicine Forense. Tomo I: Medicina Legal* (Madrid, 1969), 393-4.

36 Vincent, ‘Gender and morals’, 285.

37 Ivan Crozier and Gethin Rees, ‘Making a Space for Medical Expertise: Medical Knowledge of Sexual Assault and the Construction of Boundaries between Forensic Medicine and the Law in Late Nineteenth-Century England’, *Law, Culture and the Humanities* 8, no. 2 (2012): 285-304.

38 Uribe, *Medicina Legal*, 400; Ángel Martín de Lucenay, *Los Delitos Sexuales* (Madrid: Editorial Fenix, 1934), 11.

39 López Zan­ón and Campoy Guerrero, *Psiquiatría Forense*, 63.

40 *Ibid.*, 68.

41 *Ibid.*, 64-5, 68.

42 Hans von Hentig, ‘Remarks on the Interaction of Perpetrator and Victim’, *Journal of Criminal Law and Criminology* 31, no. 3 (1940), 303-309.

43 José Maria Reyes Monterreal, ‘Convicción Judicial por el Dictamen Médico Forense’, *Anales de Medicina Forense de la Asociación Española de Medicos Forenses* (1968-1972), 314.

44 De Alba y Osuna and Sabater Sanz, ‘El Servicio Médico Forense’, 321.

45 AHEA, C.8540; AHEA, C.1930.

46 José Augusto de Vega Ruiz, ‘Elogio de los Médicos Forenses Escrito por un Juez’, *Anales de Medicina Forense de la Asociación Española de Médicos Forenses* (September 1960), 31.

47 Some examples will be discussed below. For a discussion of such ‘boundary work’ in other contexts, see Crozier and Rees, ‘Making a Space for Medical Expertise’: 285-304.

48 See, for example, cases where victims were examined by the on-call doctor at the local community health centre (*Casa de Socorro*), AHEA, C.1930, C.15788, C.18534; by military doctors AHEA C.11257, C.17705; or by private doctors AHEA, C.17705. The military penal code stipulated that military doctors should be called upon in first assistance. In the absence of a military doctor, municipal or private doctors should be consulted. BOE 214, 2 August 1945, ‘Ley de 17 de julio de 1945 por la que se aprueba y promulga el Código de Justicia Militar (Continuación a los números 201 al 213)', art. 546.

49 Dubinsky, ‘Maidenly Girls’, 52; Stephen Robertson, ‘Seduction, sexual violence, and marriage in New York City, 1886-1955’, *Law and History Review* 24, no. 2 (2006): 347-8.

50 See Javier Bandrés and Rafael Llavona, ‘La psicologia en los campos de concentración de Franco’, *Psicothema* 8, no. 1 (1996): 1-11; Michael Richards, ‘Antonio Vallejo Nágera: Heritage, Psychiatry and War’ in *Right-Wing Spain in the Civil War Era: Soldiers of God and Apostles of the Fatherland, 1914-1945*, ed. Del Arco Blanco, M.A., and Quiroga Fernández, A. (London, 2012)*,* 195-224. During the Civil War and early post-war, Francoist scientists had been implicated in the politicisation of medicine, the most notorious example being the work of psychiatrist Antonio Vallejo Nágera to uncover the ‘bio-psychic’ roots of Marxism.

51 David Brydan, *Franco’s Internationalists: Social Experts and Spain’s Search for Legitimacy* (Oxford: Oxford University Press, 2019).

52 TMTS, 8000, leg. 105, no. 2965.

53 AGC, Caja 184, exp. 30.

54 In the policing of sexual violence, it is not unheard of for patriarchal structures to trump racial privilege. See, for example, Caitlin Cunningham, ‘The Cultural Logic of Sexual Violence in Colonial Aotearoa (New Zealand), 1842-1872’, *Journal of Women’s History* 32, no. 2 (2020): esp. 76.

55 A *careo* was often used in criminal proceedings when there was disagreement over the course of a particular event. Within rape trials this legal practice was particularly unsavoury, as (often very young) victims were forced to confront and debate their assailants as equals, with no consideration granted for the power differential between both parties.

56 TMTS, Leg. 101, no. 1807. In Spain, the Civil Guard were militarised, and therefore fell under the jurisdiction of the military courts.

57 Uribe, *Medicina Legal*, 405.

58 See, for example, Paul Preston, *The Spanish Holocaust: Inquisition and Extermination in Twentieth-Century Spain* (London: Harper Press, 2012); Julio Prada Rodríguez, *The Plundering of the Vanquished: The Economic Repression during Early Francoism* (Berlin: Peter Lang, 2019); Josefina Cuesta (ed.), *La Depuración de Funcionarios Bajo la Dictadura Franquista, 1936–1975* (Madrid: Fundación Largo Caballero, 2009).

59 Alcalde, ‘Wartime and post-war rape in Franco’s Spain’. Joly, ‘Violencias sexuadas’: 89-107; Cases Sola, ‘Violencia sexual’: 69-80. Joly and Cases Sola have argued that women's bodies constituted another front in the Spanish Civil War, and a key battleground in the reordering of Spanish society in the image of Francoism.

60 TMTS, 8000, leg 112, no, 3286. On the practice of head shaving during the Civil War, see Sánchez, *Individuas de dudosa moral*, 215-231;

61 For a discussion on the Spanish practice of pardoning, see Alcalde, ‘Wartime and post-war rape in Franco’s Spain’: 1064.

62 In fact, forensic scholars did acknowledge that non-virgins could be raped, but that this was difficult to establish via gynaecological examination, and that if force had been used there would be bruises on other parts of the body. Uribe, *Medicina Legal*, 403.

63 TMTS, 8000, 134, 4724.

64 In this sense, this work aligns itself with recent scholarship on sexual violence in conflict which has underscored the existence of many different ‘constellations’ or configurations of sexual violence beyond the ‘weapon of war’ paradigm. See, for example, Regina Mühlhäuser, 'Reframing sexual violence as a weapon and strategy of war: the case of the German Wehrmacht during the war and genocide in the Soviet Union, 1941-1944', *Journal of the History of Sexuality* 26, no. 3 (2017): 366-401. For a similar discussion in the Spanish context, see Alcalde, ‘Wartime and post-war rape’.

65 See, for example, TMTS, 8000, 134, 4724; TMTS, Leg. 109, no. 3122; AHEA, C. 2618; Archivo Histórico Provincial de Cádiz (henceforth AHPC), Abusos deshonestos, 1955, 27625, exp. 15.

66 TMTS, Causa, 663, no. 9617; Causas, leg. 651, no. 9476.

67 Here, the *forense* is referring to the work of French doctor and criminologist Henry Paul Jean Coutagne (1846-1895). Lynn Sacco has highlighted a similar readiness to dismiss the transmission of venereal disease in children in the nineteenth and twentieth century United States, pointing to the refusal of doctors to confront the prospect of incest and child abuse, particularly in ‘respectable’ families. Lynn Sacco, *Unspeakable: Father-Daughter Incest in American History* (Baltimore: John Hopkins University Press, 2009). See also, Roger Davidson, ‘“This Pernicious Delusion”: Law, Medicine and Child Sexual Abuse in Early-Twentieth-Century Scotland’, *Journal of the History of Sexuality* 10, no. 1 (2001): 69-72.

68 Crozier and Rees, ‘Making a Space for Medical Expertise’.

69 Uribe, *Medicina Legal*, 411.

70 ‘Dishonest abuse’ was the term used to refer to sexual abuse falling short of vaginal penetration by the penis.

71 AHEA, C.15788.

72 This allusion to the sexual deviance of those with intellectual disabilities reflected broader international trends over the perceived ‘animalistic’ instincts of such individuals. See Joanna Bourke, '"Animal instincts": the sexual abuse of women with learning difficulties, 1930s-1910s', Women's History Review (2020); Louise Hide, 'In Plain Sight: Open Doors, Mixed-sex Wards and Sexual Abuse in English Psychiatric Hospitals, 1950s to Early 1990s', *Social History of Medicine*, 31, no. 4 (2018): 732-53.

73 AHEA, C.13316.

74 Bourke, ‘Sexual Violence, Bodily Pain, and Trauma’: 25-51.

75 Archivo General de la Administración, Audiencia Territorial de Madrid (henceforth ATM), Caja 200, Estupro, 1941.

76 On cinemas and other public spaces as locations for clandestine erotic activity under Francoism, see Rom­án Ruiz, ‘¿El baile agarrado es pecado?’: 319-228.

77 Vincent, ‘Gender and morals’: 284-6.

